# Study on Combustion Kinetic Characteristics of Lignite and Semi-coking Dust

**DOI:** 10.1021/acsomega.2c03199

**Published:** 2022-09-06

**Authors:** Jiaqi Yu, Di Sha

**Affiliations:** College of Safety and Emergency Management, Taiyuan University of Technology, Taiyuan 030024, China

## Abstract

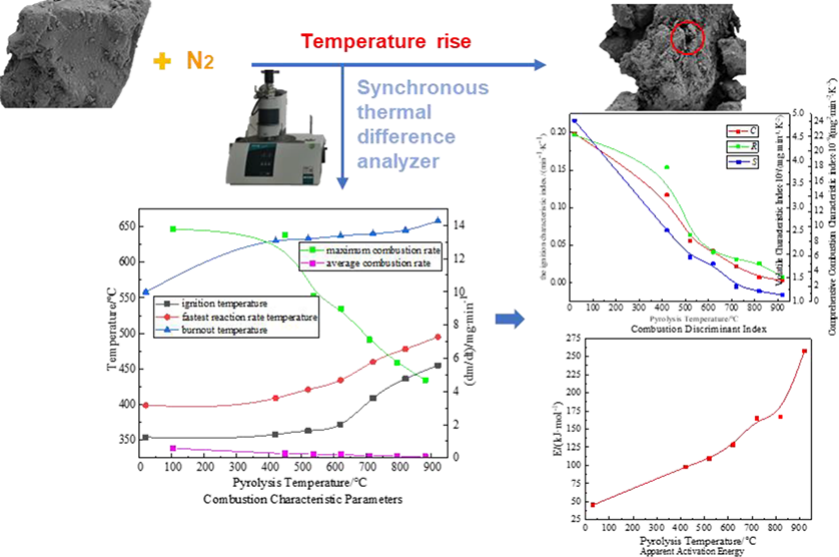

In order to master the combustion kinetic characteristics of semi-coking dust in the early pyrolysis stage of lignite combustion explosion, a vacuum tube furnace was used to prepare semi-coking dust with different pyrolysis degrees, and the experimental samples were studied by a synchronous differential thermal analyzer. By means of theoretical analysis, the reaction mechanism of lignite and semi-coking dust was revealed. The results show that when the final pyrolysis temperature rises to 920 °C, the percentage of volatile matter decreases by 94.6%. The reaction in this process also causes the original pores to be cross-linked and collapsed, and a large number of new pores are generated, and the original pore structure is significantly enlarged. With the increase of the final temperature of pyrolysis, the ignition temperature (*T*_b_) of the dust increased from 354 to 455 °C, the fastest reaction temperature (*T*_c_) increased from 399 to 495 °C, and the ember temperature (*T*_d_) increased from 558 to 658 °C. The maximum combustion rate decreased by 65.97%, and the average combustion rate decreased by 84.67%. The apparent activation energy increased by 4.7 times from 45.219 to 257.665 kJ/mol, and the combustion kinetics of semi-coke became worse. The thermal reaction of lignite and semi-coking dust conforms to the diffusion mechanism of the three-dimensional spherical symmetry model. The research results provide a new idea for discussing the mechanism of coal dust explosion and the development of explosion suppression technology.

## 1 Introduction

China has always been the largest coal producer in the world.^1^ At present, more than 95% of coal mining enterprises adopt the production mode of underground well work. A large amount of coal dust is often produced in the process of coal mine production and processing. Once these coal dust combustion and explosion accidents occur, it will cause heavy casualties and property losses to the front-line construction workers underground. Therefore, it is particularly important to ensure the safe production of underground coal mine.

According to the mechanism of coal dust explosion, it can be known that in the initial stage of coal dust explosion, coal particles are heated to undergo pyrolysis reaction, and a large amount of combustible volatile gas is precipitated. This is also the essential reason affecting the occurrence of coal dust explosion.^2^ At present, a lot of research has been done on the pyrolysis reaction of coal at home and abroad.

Some scholars^3−7^ studied the evolution of chemical structure of coal during pyrolysis and found that the structure of semi-coke is the main factor affecting combustion performance. Wang et al.^8,9^ analyzed the pyrolysis characteristics of coal through experimental research. Zhao et al.^10−12^ proved through experiments that the synergistic effect of physical mixed co-pyrolysis has an obvious effect on the pyrolysis products. Taking lignite and corn straw as the research objects, they studied the feasibility of catalytic reforming of the co-pyrolysis volatiles of three iron ores. Tetsuya Fukuoka et al.^9^ analyzed the solid chemical structure and gaseous distribution of coal under two same pyrolysis conditions, which supplemented the coal pyrolysis (CP) mechanism. Mishra et al.^14,15^ revealed the evolution path of coal structure pyrolysis reaction at different temperatures and thus proposed the devolatilization mechanism of chemical structure evolution in coal high temperature pyrolysis reaction. Some researchers analyze the characteristics and mechanism of CP through multi-scale simulation, so as to realize the sustainable development of coal industry. Bayartsaikhan et al.^16^ accurately predicted the amount of final volatile matter based on the kinetic parameters obtained during pyrolysis, combined with the reaction model. Some researchers used ReaxFF molecular dynamics (MD) simulation to study the comprehensive dynamic migration mechanism of organic oxygen in coal particle pyrolysis reaction, which provides a new method to study the overall behavior and complex mechanism of different coal rank pyrolysis.^17,18^ Yan et al.^19,20^ used the distributed activation energy model (DAEM) to correlate the carbon structure, pyrolysis characteristics, and kinetic characteristics of 4 middle-low rank coals. The results show that DAEM can accurately predict the pyrolysis behavior of bituminous coal at high temperature rise rate, but it is less effective in lignite pyrolysis modeling and prediction. Cui et al.^21^ proposed a clean system integrating CP and chemical cycle gasification process by using multi-scale modeling methods such as MD simulation, computational fluid dynamics simulation, and process simulation, so as to realize the sustainable development of coal industry.

At present, a lot of research has been done on the reaction mechanism of CP. By combining the experimental results with numerical simulation, the coal was modified and upgraded to achieve the purpose of large-scale utilization. However, the lack of research on the thermal analysis kinetic characteristics and combustion characteristics of intermediates in the initial pyrolysis process of coal dust explosion at the initial stage limits the development of explosion suppression technology. Lignite has the highest volatile content, strong chemical reaction, poor thermal stability, and low thermal safety.^22,23^ Therefore, in this study, lignite was selected and pyrolyzed at high temperature. By controlling different final pyrolysis temperatures, different degrees of semi-coking dust in the pyrolysis stage before explosion were simulated and prepared. The differences in industrial analysis indexes, micro-morphological characteristics, and combustion kinetic characteristics of lignite and semi-coking dust were explored, and the thermal reaction mechanism of lignite and semi-coking dust was revealed through comprehensive theoretical research. The research results have important guiding significance for improving the occurrence mechanism of coal dust explosion, taking reasonable and effective explosion suppression measures, and developing explosion suppression products and technologies.

## 2 Materials and Methods

### 2.1 Experimental Sample Preparation

#### 2.1.1 Preparation of Lignite Dust

Due to the abundant lignite resources in Inner Mongolia, China, the experimental samples of this study were selected from lignite in Inner Mongolia. After the successful collection of coal samples, the experimental samples were immediately put into the coal sample tank for sealed storage to prevent the experimental samples from being polluted or oxidizing in contact with oxygen in the air. When preparing the sample, a small amount of coal sample should be taken out and placed in an agate bowl, which was manually ground for 200 mesh. After grinding, place it in a sealed bag for standby.

#### 2.1.2 Preparation of Semi-coking Dust of Lignite

The semi-coking dust of lignite is the product of volatiles released in the pyrolysis stage of lignite dust in the early stage of combustion. Therefore, the ground lignite dust was put into the crucible, and a vacuum tube furnace was used to heat the crucible containing the coal samples to obtain different degrees of semi-coking dust in the lignite pyrolysis stage. In order to prevent the oxidation combustion reaction of coal dust when heated, inert gas N_2_ was introduced into the whole heating process. The gas flow rate was 100 mL/min, and the heating rate was 10 °C/min. Under this condition, lignite began to react chemically when heated to 420 °C and produce combustible gas. When the heating temperature reached 920 °C, the volatile gas in coal dust could be completely separated out.^2,24,25^ Therefore, in the process of preparing the experimental sample, the final heating temperature started at 420 °C and gradually increased in steps of 100 °C. The semi-coking dust with final pyrolysis temperatures of 420, 520, 620, 720, 820, and 920 °C was prepared and kept at a constant temperature for 20 min. After that, it can be naturally cooled to room temperature and the experimental sample can be taken out.

#### 2.1.3 Industrial Index Parameters of Lignite and Semi-coking Dust

An industrial analysis of the lignite and semi-coking were conducted in accordance with GB/T 212-2008^25^ “Proximate analysis of coal” and GB/T 31391-2015^26^ “Elemental analysis of coal”. The results of proximate analysis and elemental analysis are given in Table 1.

**Table 1:** Proximate Analysis and Elemental Analysis Results of Lignite and Semi-coking Dust/%^a^

	proximate analysis of coal				elemental analysis of coal				
sample	*V*daf/%	*M*ad/%	*A*ad/%	FCad/%	C	H	O	N	S_t_
lignite	45.4	1.24	8.07	45.29	71.97	4.52	21.04	1.21	1.26
420 °C pyrolysis	27.53	0.27	8.41	63.79	73.35	4.01	19.65	1.57	1.42
520 °C pyrolysis	18.21	0.19	8.82	72.78	76.58	3.49	17.42	1.60	0.91
620 °C pyrolysis	15.17	0.14	9.33	75.36	79.29	2.81	15.51	1.52	0.87
720 °C pyrolysis	10.2	0.12	10.62	79.06	82.84	2.10	12.92	1.40	0.74
820 °C pyrolysis	4.16	0.09	10.98	84.77	90.03	1.83	6.09	1.37	0.68
920 °C pyrolysis	2.45	0.07	11.37	86.11	93.68	1.19	3.14	1.35	0.64

a *V*_daf_ is the percentage of dry ash-free volatile matter, *M*_ad_ is the percentage of moisture, *A*_ad_ is the percentage of ash, and FC_ad_ is the percentage of fixed carbon.

As can be seen from Table 1, with the increase of pyrolysis final temperature, the percentage content of volatile matter, moisture, H, O, and S elements in semi-coking dust gradually decreases. This is because with the increase of temperature, water in lignite evaporates, and lignite structure contains a large number of oxygen-containing functional group structures, resulting in pyrolysis reaction. The oxygen-containing functional groups and alkyl side chains in coal are separated out in the form of volatiles and are gradually consumed. Therefore, the relative percentage of fixed carbon and ash in semi-coking dust gradually increases. The nitrogen in coal mostly exists in the form of more stable pyrrole and pyridine; therefore, the nitrogen content changes little.^2^

#### 2.1.4 Surface Morphology Characteristics of Lignite and Semi-coking Dust

**Figure 1:**
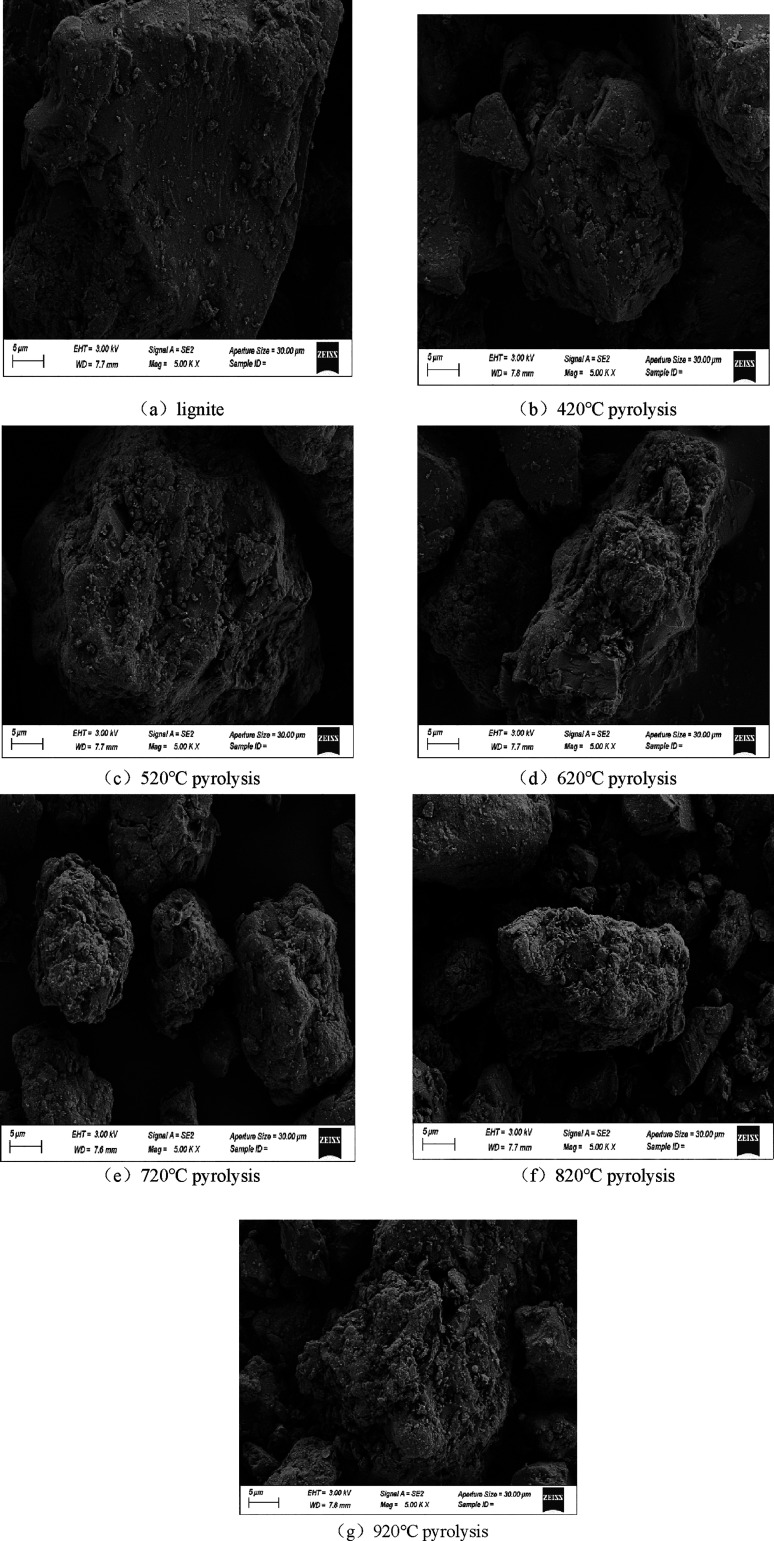
Morphological changes of lignite surface under different final pyrolysis temperatures.

In order to observe the micro-morphological characteristics of semi-coking dust after high-temperature pyrolysis of lignite and precipitation of volatile matter, it was observed under the condition of 5000 times magnification of scanning electron microscope. The results are shown in Figure 1a–g.

The surface of lignite is relatively flat without obvious pore structure. With the increase of the final temperature of pyrolysis, the surface structure of semi-coking dust became more and more looser and the pores became more obvious. During the heating process of coal particles, pyrolysis reaction will occur, so that the solid organic matter in the coal structure is consumed and precipitated in the form of volatile gas. In this process, the original pore structure was greatly changed due to the effect of surface tension. The original closed pores were opened and enlarged. With the polycondensation reaction, a large number of new pores were also generated. When the final pyrolysis temperature was 920 °C, a large amount of volatiles were precipitated, and the cross-linking and collapse of pores directly led to the reduction of micropores and the increase of macropores, so the pores on the particle surface are obvious.

### 2.2 Thermogravimetric Experimental Analysis Method of Lignite and Semi-coking Dust

**Figure 2:**
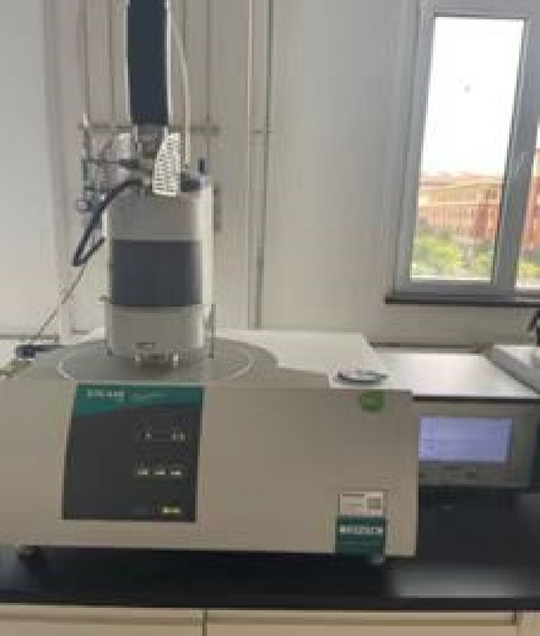
Synchronous thermal analyzer.

The synchronous thermal analyzer was used in this test. The experimental device is shown in Figure 2.

The test experimental conditions were set in the mixed gas atmosphere with O_2_/N_2_ of 1:4. Take 10 mg of experimental sample (lignite and semi-coking dust) at a heating rate of 10 °C/min, conduct comprehensive thermal analysis test during heating to 800 °C, and then draw the corresponding isothermal TG curve.

### 2.3 Theoretical Analysis Method of Lignite and Semi-coking Dust

#### 2.3.1 Combustion Kinetics Analysis Method of Lignite and Semi-coking Dust

##### 2.3.1.1 Ignition Characteristic Index (*C*)

The ignition characteristic index can evaluate the difficulty of the ignition process of lignite and semi-coking dust. The greater its value, the lower the ignition conditions of the experimental samples.^27^ Calculate with formula 1.
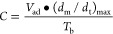
1where *C* is the ignition characteristic index of dust (mg•min^–1^•K^–1^), *V*_ad_ is the percent volatile content (%), (*d*_m_/*d*_t_)_max_ is the maximum rate of combustion reaction (mg•min^–1^), and *T*_b_ is the ignition temperature under the condition of dust accumulation (°C).

##### 2.3.1.2 Volatile Precipitation Characteristic Index (*R*)

The precipitation process and capacity of volatile matter play an important role in the combustion of lignite and semi-coking dust. In order to evaluate the precipitation of volatile matter during the heating process of lignite and semi-coking dust,^28^formula 2 is used.
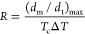
2where *R* is dust volatile precipitation characteristic index (mg•min^–1^•K^–2^), *T*_c_ is the peak temperature (°C), and Δ*T* is the temperature range between peak temperature and ignition temperature (°C), Δ*T* = *T*_c_ – *T*_b_.

##### 2.3.1.3 Comprehensive Combustion Characteristic Index (*S*)

The comprehensive combustion characteristic index (*S*) is used to comprehensively evaluate the dust combustion characteristics,^29^ and the calculation process is shown in formula 3.
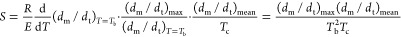
3where *S* is the comprehensive combustion characteristic index (mg^2^•min^–2^•K^–3^), *R* is expressed as the gas reaction constant (8.314 × 10^–3^ kJ•mol^–1^•K^–1^), *E* is the apparent activation energy (kJ/mol), (*d*_m_/*d*_t_)_*T*=*T*_*i*__ is the combustion rate at the time of ignition temperature (mg/min), (*d*_m_/*d*_t_)_mean_ is the average combustion rate (mg/min), and *R*/*E* is the reactivity of dust. The smaller the activation energy, the greater the reaction activity.  is the conversion of combustion rate at ignition temperature, indicating the reaction degree of ignition. Each index comprehensively reflects the ignition and combustion characteristics of dust. The greater the *S* value, the more likely the dust is to catch fire and its combustion performance is stable, which is also more conducive to the spread of flame.

##### 2.3.1.4 Apparent Activation Energy of Dust

The minimum energy required for dust to undergo a combustion chemical reaction is the apparent activation energy, and the activation energy is different in different chemical reaction processes. Thermal analysis kinetics is a method to determine the reaction-related kinetic parameters by using thermal analysis technology based on the thermal analysis kinetic equation. It obeys Arrhenius law,^30^ and its basic dynamic equation is 4
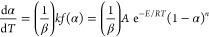
4where β is the heating rate (°C/min), *k* is the reaction rate constant, α is the conversion rate (%), *f*(α) is the reaction mechanism function, and *A* is the pre factor, also known as pre factor or frequency factor (s^–1^).

According to the thermogravimetric curve, the conversion rate of dust in the process of heating reaction can be obtained as α.^31^Formula 5 can be used for calculation
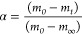
5where *m*_*t*_ is the mass of dust at time *t*, *m*_0_ is the initial mass of dust, and *m*_∞_ is the mass after dust reaction.

Because the apparent activation energy of dust at the same heating rate under non-isothermal conditions was considered in this study, set the combustion reaction of dust as the first-order reaction, that is, *n* = 1. The Coats–Redfern method was used for analysis and research, and formula 6 was obtained
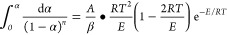
6

Take logarithms on both sides at the same time to obtain formula 7
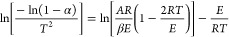
7

According to eq 7,  is a straight line whose independent variable is1/*T*, the slope is approximately −*E*/*R*, and the intercept is ln[*AR*/β*E*(1 – 2*RT*/*E*)]. Therefore, the weight loss rate of the experimental sample is read out in the TG curve, and according to the conversion rate α,  and 1/*T* can be calculated accordingly. The activation energy *E* of dust is further calculated.

#### 2.3.2 Analysis Method of Heating Reaction Mechanism of Lignite and Semi-coking Dust

The reaction mechanism function, as the reaction kinetic mechanism function equation, reveals the functional relationship between the reaction rate constant *k* of the solid substance and the conversion rate α. Then, describe the chemical reaction process of material molecules. The reaction mechanism models of lignite and semi-coking dust in pyrolysis and oxidation process mainly include random nucleation model, reaction order model, diffusion mechanism model, and phase interface reaction model. Table 2 shows commonly used and recognized solid-state reaction kinetic models.^32^
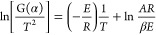
8
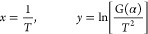

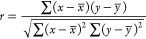
9

**Table 2:** Commonly Used Kinetic Model Functions of Solid State Reactions

mode code	differential form: *f*(α)	integral form: G(α)	reaction model
A_1_	1 – α	–ln(1 – α)	random nucleation model
A_2_	2(1 – α)[1 – ln(1 – α)^1/2^	[−ln(1 – α)]^1/2^	random nucleation model
A_3_	3(1 – α)[1 – ln(1 – α)^2/3^	[−ln(1 – α)]^1/3^	random nucleation model
A_4_	4(1 – α)[1 – ln(1 – α)^3/4^	[−ln(1 – α)]^1/4^	random nucleation model
D_1_	α/2	α^2^	one-dimensional diffusion mechanism model
D_2_	[−ln(1 – α)]^−1^	(1 – α) ln(1 – α) + α	two-dimensional diffusion mechanism model
D_3_	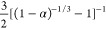	(1 – 2/3α) – (1 – α)^2/3^	three-dimensional diffusion mechanism model (cylindrical symmetry)
D_4_	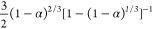	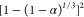	three-dimensional diffusion mechanism model (spherical symmetry)
F_2_	(1 – α)^2^	(1 – α)^−1^ – 1	reaction order model *n* = 2
F_3_	(1 – α)^3^	1/2[(1 – α)^−2^ – 1]	reaction order model *n* = 3
R_2_	2(1 – α)^1/2^	1 – (1 – α)^1/2^	phase interface reaction model
R_3_	3(1 – α)^2/3^	1 – (1 – α)^1/3^	phase interface reaction model

Substitute various reaction mechanism functions *G*(α) in Table 2 into formula 8, process the thermogravimetric curve data of lignite and semi-coking dust, and calculate the conversion rate α under different temperatures. The conversion rate α is substituted into the reaction mechanism function *G*(α), and the linear curve of ln[*G*(α)/*T*^2^] and 1/*T* is obtained by linear fitting according to formula 9 to calculate the fitting correlation coefficient *r*. The closer |*r*| is to 1, the better the correlation coefficient is. The best correlation coefficient represents the reaction mechanism function of dust under non-isothermal conditions.

## 3 Results and Discussion

### 3.1 Thermogravimetric Curve Analysis

**Figure 3:**
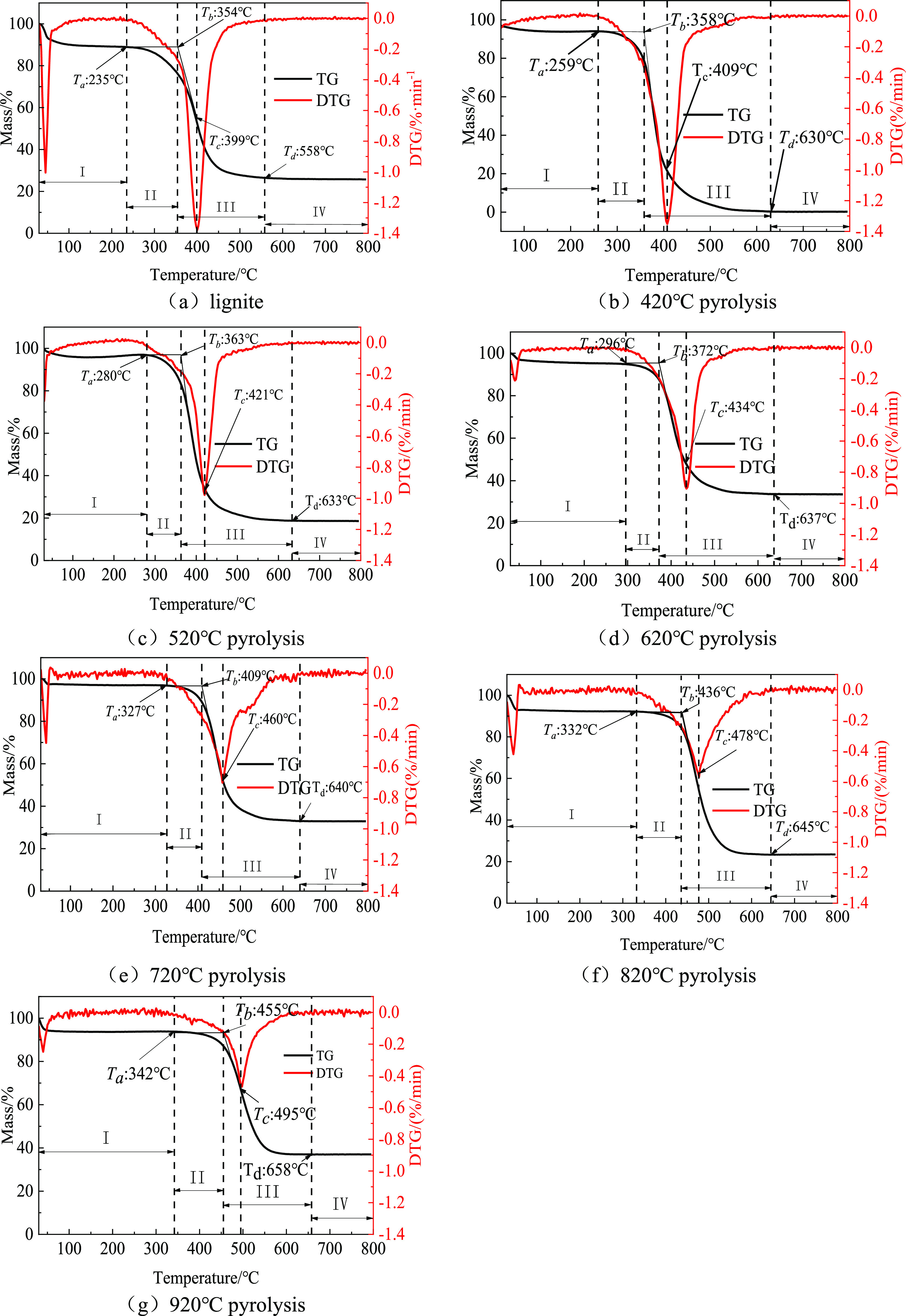
TG-DTG curves of lignite and semi-coking dust.

According to the TG-DTG curve of lignite and semi-coking dust during heating (Figure 3a–g), the whole reaction process can be divided into four stages. The first stage is the process of heating dehydration and oxygen absorption weight gain of the experimental sample. The second stage is the process in which the experimental sample is heated to release volatile gas. The third stage is the combustion reaction process of the experimental sample. The fourth stage is the burnout stage of the experimental sample.

During the experiment, the samples were heated from 30 °C, and the experimental samples began to enter the weight loss stage. At the initial stage of heating, the weight loss of the sample is obvious. Because coal is a porous medium structure, it is very easy to adsorb water and gas. Therefore, the first stage is mainly the process of weight loss of free water and adsorbed gas in the experimental sample and weight gain by oxygen inhalation. At this stage, due to the increase of temperature, the water in the sample evaporates first, and then, the active organic functional groups in the coal will react with the oxygen in the air to promote the quality to increase. When the temperature reaches *T*_a_, the mass is the maximum. Continue to raise the temperature. When the mass of water loss and volatile matter is greater than the mass of oxygen absorption, the mass of the experimental sample will decrease and begin to enter the second stage (*T*_a_–*T*_b_), in which the experimental sample is heated to release volatile gas. *T*_b_ is the ignition temperature of the experimental sample in the stacking state. The ignition temperature was determined by the “tangent method”. First, find out the maximum temperature point *T*_c_ of the reaction rate. By making a vertical line to the peak point on the DTG curve, the temperature point corresponding to the point intersecting the TG curve was the temperature point of the fastest reaction rate. Make a tangent line on the TG curve through this temperature point, and the baseline intersection with the highest point *T*_a_ of oxygen absorption and weight gain after complete dehydration of the coal sample is the ignition temperature point of the experimental sample in the stacking state. Further increasing the temperature, the sample will enter the third stage, the combustion reaction stage. With the increase of heating time, the mass of the experimental sample first decreases slowly, then decreases in a cliff-like manner, and then remains stable. This rapid decline process is also the high-temperature combustion stage of the experimental sample. In this process, the dust particles are heated, which leads to the reaction of organic functional groups with high activity in the structure and releases a large amount of volatile gas. These flammable volatile gases and the organic matter that is difficult to volatilize together undergo a combustion reaction under the action of a high-temperature heat source and release a large amount of heat to ignite the dust. When the temperature rises to *T*_c_, the reaction rate reaches the highest value and the combustion reaction is the most intense. Continue to increase the temperature. When the temperature reaches *T*_d_, the heating mass of the experimental sample does not change; it will enter the fourth stage, that is, the burning ember stage. At this time, the residue in the sample is ash and other non-combustible impurities.^33^

From the weight loss rate of the sample in DTG curve, it can be seen that the whole combustion reaction is mainly divided into two parts.^34^ When the temperature is lower than 100 °C, there is a peak. At this stage, the gas and free water absorbed in the coal particles are heated and precipitated, which is a physical reaction process. When the temperature reaches *T*_c_, the maximum weight loss rate is reached, and the combustion is the most intense at this stage. In this reaction stage, it is mainly the ignition and combustion of the combustible volatile gas and the organic matter in the dust particles that produces a lot of heat at the same time, and then leads to the combustion of fixed carbon. It is a chemical reaction process.

The ignition temperature (*T*_b_), the fastest reaction rate (*T*_c_), the ember temperature (*T*_d_), and the maximum combustion rate of lignite and semi-coking dust can be clearly seen from the TG-DTG curve in Figure 3. Through calculation, we can know the average combustion rate in the whole reaction process. The results are shown in Table 3 to analyze the combustion characteristics of lignite and semi-coking dust.

**Table 3:** Combustion Characteristic Parameters of Lignite and Semi-coking Dust

sample	*T*b/°C	*T*c/°C	*T*d/°C	(*d*_m_/*d*_t_)_max_/mg•min^–1^	(*d*_m_/*d*_t_)_mean_/mg•min^–1^
lignite	354	399	558	13.78	0.574
420 °C pyrolysis	358	409	630	13.45	0.254
520 °C pyrolysis	363	421	633	9.75	0.223
620 °C pyrolysis	372	434	637	9.01	0.212
720 °C pyrolysis	409	460	640	7.12	0.118
820 °C pyrolysis	436	478	645	5.72	0.11
920 °C pyrolysis	455	495	658	4.69	0.088

It can be seen from Table 3 that the ignition temperature (*T*_b_), the fastest reaction rate temperature (*T*_c_), and the burnout temperature (*T*_d_) of semi-coking dust of lignite in the later stage of pyrolysis reaction gradually increase, and the reaction is more difficult. The maximum combustion rate ((*d*_m_/*d*_t_)_max_) and average combustion rate ((*d*_m_/*d*_t_)_mean_) reflect the intensity of the combustion reaction process of lignite and semi-coking dust. The reaction intensity of semi-coking dust gradually decreases with the increase of final pyrolysis temperature. Lignite has high volatile content, so it is unstable and prone to reaction with high reaction intensity. After high-temperature pyrolysis, the higher the final temperature of pyrolysis, the lower the volatile content of the semi-coking dust obtained. The ignition temperature, the most intense reaction temperature point, and the burnout temperature point all move backward. The more difficult it is to have combustion reaction, the reaction rate decreases and the reaction intensity gradually weakened.

### 3.2 Combustion Kinetics Analysis of Lignite and Semi-cooking Dust

Based on the analysis of the combustion characteristic parameters of lignite and semi-coking dust in Table 3, the combustion discrimination index of lignite and semi-coking dust is obtained. The calculation results are shown in Table 4.

**Table 4:** Combustion Discriminant Index of Lignite and Semi-coking Dust

sample	*C*/min^–1^•K^–1^	*R* × 10^–4^/mg•min^–1^•K^–2^	*S* × 10^–9^/mg^2^•min^–2^•K^–3^
lignite	0.199	4.55	24.09
420 °C pyrolysis	0.117	3.86	9.46
520 °C pyrolysis	0.056	2.42	5.90
620 °C pyrolysis	0.042	2.05	5.02
720 °C pyrolysis	0.021	1.9	1.97
820 °C pyrolysis	0.007	1.81	1.36
920 °C pyrolysis	0.003	1.52	0.83

The ignition characteristic index (*C*) is used to indicate how easily combustible dust ignites. The larger the ignition characteristic index, the easier combustible dust is to ignite, and the better the ignition characteristic. Volatile characteristic index (*R*) is an index to evaluate the performance of volatile analysis. The higher the volatile characteristic index is, the easier the volatile is to be precipitated. The comprehensive combustion characteristic index (*S*) is a comprehensive index to evaluate the ignition and combustion of combustible dust. The greater the comprehensive combustion characteristic index, the lower the difficulty of ignition of combustible dust, the more stable the combustion performance, and the more conducive the stable propagation of flame. It can be seen from Table 4 that with the increase of final pyrolysis temperature, the ignition characteristic index of semi-coking dust gradually decreases, and the more difficult it is to be ignited. However, the content of residual volatile in semi-coking dust is less, and the more difficult it is to precipitate, resulting in the gradual decrease of precipitation rate. This is also related to the structural components of semi-coking dust. The comprehensive combustion characteristic index of semi-coking dust gradually decreases with the increase of final pyrolysis temperature of lignite dust. The less easily semi-coking dust ignites, the more unfavorable it is to the stable propagation of flame. This is because the coal first precipitates volatiles during the heating process and fires, followed by coke combustion reaction. Therefore, volatile matter has an important influence on the ignition performance of coal. The higher the volatile matter, the better the ignition performance. During the heating process of lignite dust, the volatile matter in coal is continuously separated out. Thus, the residual volatile matter becomes less, resulting in the decrease of ignition performance and comprehensive combustion performance. In addition, the ash shell formed on the surface of lignite dust will hinder the contact between the combustible volatile gas precipitated during pyrolysis and oxygen, thus reducing the concentration of oxygen in the combustion reaction process. At the same time, it will also hinder the heat transfer between unburned coal particles and the surrounding environment, and affect the combustion and burning of semi-coking dust. Therefore, the ash content in lignite dust and semi-coking dust also has an important influence on their combustion characteristics. With the increase of final pyrolysis temperature, the ash content in semi-coking dust increases significantly. The higher the ash content, the lower the ignition performance and comprehensive combustion characteristics.

**Figure 4:**
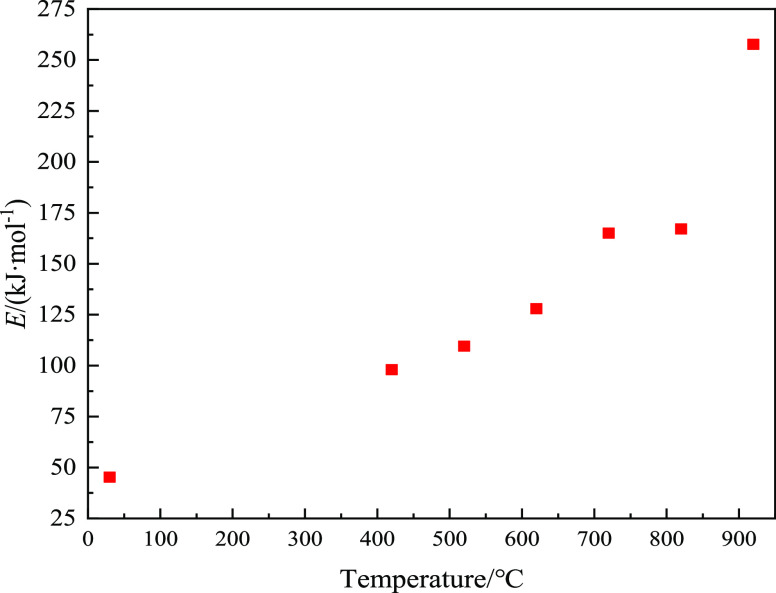
Apparent activation energy variation of dust particles from lignite and semi-coking dust.

Fit the data, and calculate the apparent activation energy in the dust reaction process by fitting the curve data. The results are shown in Table 5, and the change rule is shown in Figure 4.

**Table 5:** Apparent Activation Energy of Lignite and Semi-coking Dust

sample	–*E*/*R*	*E*/kJ•mol^–1^	ln[*AR*/β*E*(1 – 2*RT*/*E*)]	*R*^2^
lignite	–5438.89	45.219	–3.726	0.9311
420 °C pyrolysis	–11,785.88	97.994	3.298	0.8936
520 °C pyrolysis	–13,171.92	109.518	5.349	0.9125
620 °C pyrolysis	–15,384.61	127.915	10.370	0.9597
720 °C pyrolysis	–19,842.16	164.978	18.354	0.9133
820 °C pyrolysis	–20,089.77	167.026	17.914	0.8722
920 °C pyrolysis	–30,989.83	257.665	31.340	0.9294

Generally speaking, when the activation energy is in the range of 40–400 kJ•mol^–1^, it is considered that combustible dust can undergo combustion chemical reaction. When the activation energy value is less than 40 kJ•mol^–1^, the smaller the minimum energy that can cause the reaction, the faster the reaction, which is basically completed in an instant. With the increase of activation energy, the energy required for the reaction increases, the reaction rate decreases, and the reaction difficulty increases. When the activation energy value is greater than 400 kJ•mol^–1^, the greater the energy required to cause the reaction, and the reaction rate is very slow. It can be basically determined that the chemical reaction cannot occur.^35^

It can be seen from Table 5 that the apparent activation energy values of all experimental samples are between 45 and 258 kJ•mol^–1^; that is, chemical reactions occur. However, the apparent activation energy of semi-coking dust in different pyrolysis stages is obviously different, and the apparent activation energy increases rapidly with the increase of pyrolysis temperature. In other words, lignite is relatively easy to react, which is also reflected in the analysis of the ignition performance of lignite and semi-coking dust. In essence, the apparent activation energy of dust shows an obvious change rule with the content of volatile matter in dust. As the volatile matter of lignite dust is relatively high, more combustible gases are released in the pyrolysis process and the reaction activity is relatively strong. With the decrease of volatile content in semi-coking dust, the combustible gas that can be precipitated during pyrolysis also decreases, the combustion performance weakens, the apparent activation energy increases significantly, the reaction rate during chemical reaction slows down accordingly, and the macro-reaction is relatively difficult.

### 3.3 Analysis of Heating Mechanism of Lignite and Semi-coking Dust

The Coats–Redfern method was used to solve the thermal reaction mechanism functions of lignite and semi-coking dust. The correlation coefficients |*r*| of various reaction mechanism functions of lignite and semi-coking dust were obtained, and the results are shown in Table 6.

**Table 6:** Correlation Coefficients of Dust Reaction Mechanism Functions

mode code	lignite	420 °C pyrolysis	520 °C pyrolysis	620 °C pyrolysis	720 °C pyrolysis	820 °C pyrolysis	920 °C pyrolysis
A_1_	–0.9966	–0.9787	–0.9844	–0.99	–0.9733	–0.99	–0.996
A_2_	–0.9829	–0.9966	–0.9979	–0.9902	–0.9978	–0.9965	–0.9968
A_3_	–0.9958	–0.9979	–0.9897	–0.9925	–0.9952	–0.9971	–0.9955
A_4_	–0.9971	–0.9957	–0.9921	–0.9957	–0.9981	–0.9969	–0.9969
D_1_	–0.9954	–0.9931	–0.9953	–0.9979	–0.9959	–0.9981	–0.9928
D_2_	–0.9942	–0.9975	–0.9982	–0.9967	–0.9975	–0.9939	–0.9943
D_3_	–0.9959	–0.9963	–0.9969	–0.9965	–0.99	–0.9942	–0.9947
D_4_	–0.9995	–0.9992	–0.9989	–0.999	–0.9986	–0.9983	–0.9971
F_2_	–0.9951	–0.9939	–0.9945	–0.9922	–0.9964	–0.9979	–0.9962
F_3_	–0.9931	–0.9965	–0.9935	–0.9975	–0.9969	–0.9966	–0.9958
R_2_	–0.9946	–0.9943	–0.9971	–0.9983	–0.9971	–0.9948	–0.9938
R_3_	–0.9943	–0.9989	–0.9985	–0.9968	–0.9965	–0.9921	–0.9945

According to Table 6, the correlation coefficient |*r*| of the reaction function whose mode code D_4_ is the closest to 1, and the correlation is the best. That is
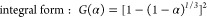
10
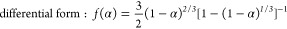
11

Therefore, the thermal reaction mode of lignite and semi coking dust is more in line with the three-dimensional (spherical symmetry) diffusion mechanism model. The particle surface contacts the heat source and decomposes when heated to release combustible volatile gas, which diffuses from the particle surface to the surrounding.

## 4 Conclusions

In this paper, the dynamic characteristics of lignite and semi-coking dust combustion process were analyzed by combining experimental research and theoretical analysis, and the heating reaction mechanism of lignite and semi-coking dust was revealed. The main conclusions are as follows:

(1) The industrial analysis indexes of lignite and semi-coking dust were analyzed, and the surface morphology of lignite and semi-coking dust was observed. With the increase of the final pyrolysis temperature, the water content decreases, and the coal structure reacts when heated and is separated out in the form of volatile, so that the relative percentage content of volatile is reduced. The relative percentage content of water also decreases correspondingly. The relative percentage of ash and fixed carbon increased significantly. The reaction in this process also directly leads to the generation of new pores, and the original pores will also be cross-linked and collapsed, so the pore structure is also obviously enlarged.
(2) The thermogravimetric curves and combustion kinetics of lignite and semi-coking dust were analyzed. With the increase of the final pyrolysis temperature, the combustion characteristic indexes of lignite and semi-coking dust increase. According to the combustion discrimination index, the higher the final pyrolysis temperature, and the lower the combustion discrimination index of lignite and semi-coking dust, the less likely it is to react and the more difficult it is to ignite. With the increase of final temperature of pyrolysis, the apparent activation energy increases significantly, with an average increase of 38.29%, resulting in more difficult reaction, which corresponds to the combustion discrimination index characteristics of lignite and semi-coking dust.
(3) The thermal reaction mechanism of lignite and semi-coking dust was analyzed, according to the commonly used solid-state reaction kinetic model function to solve the thermal decomposition reaction mechanism function of lignite and semi-coking dust. Through the fitting correlation coefficient, it is determined that the reaction mode is more in line with the three-dimensional (spherical) symmetry of the diffusion mechanism model. The dust reaction mechanism mode is revealed; that is, the particle surface contacts the heat source, the combustible volatile gas is released by thermal decomposition, and the gas diffuses from the particle surface to the surrounding.

Based on experiments, this study studied the basic characteristics and combustion kinetic characteristics of lignite and semi-coking dust and revealed the thermal reaction mechanism of lignite and semi-coking dust by theoretical analysis. The results have important basic significance for taking effective explosion-proof and explosion suppression measures in the initial pyrolysis stage of coal dust explosion. However, under the current experimental conditions, the semi-coking dust can only approximate the pyrolysis products in the early stage of explosion. On the basis of this study, a fast pyrolysis device experimental platform simulating the real pyrolysis reaction in the early stage of explosion will be built, close to the heating rate of the pyrolysis reaction in the early stage of explosion, and the real semi-coking dust in the early stage of explosion will be produced for explosion suppression technology and theoretical research.

## Notes

The data used to support the findings of this study are available from the corresponding author upon request.
